# A Popular Treatise on the Skin and Hair

**Published:** 1854-10

**Authors:** 


*A Popular Treatise on the Skin and Hair, their Preservation and Management.* By ERASMUS WILSON, F. R. S.. Author of a Treatise "on Diseases of the Skin," A System of Human Anatomy," &c. &c. Second American, from the fourth and revised London edition; with illustrations. Philadelphia: Blanchard and Lea, 1854

Former editions of this work have been before the public for a considerable time, and its merits are consequently well known. As its title imports, it is designed for the *general* rather than the professional reader, and is well calculated to impart knowledge of much value to all classes.

